# Inverted internal limiting membrane flap technique in eyes with large idiopathic full-thickness macular hole: long-term functional and morphological outcomes

**DOI:** 10.1007/s00417-021-05082-7

**Published:** 2021-01-29

**Authors:** Nathalie Bleidißel, Julia Friedrich, Julian Klaas, Nikolaus Feucht, Chris Patrick Lohmann, Mathias Maier

**Affiliations:** 1 Department of Ophthalmology, Klinikum rechts der Isar, Technical University Munich (TUM), Ismaningerstr. 22, 81675 Munich, Germany; 2 Smile Eyes Augenklinik Airport, Munich, Germany

**Keywords:** Inverted internal limiting membrane flap technique, Large macular hole, Spectral-domain optical coherence tomography, External limiting membrane, Flap appearance, Macular hole index

## Abstract

**Purpose:**

To investigate morphological and functional outcomes of the inverted internal limiting membrane (I-ILM) flap technique in large (≥ 400 μm) idiopathic full-thickness macular holes (FTMH) over a follow-up period of 12 months.

**Methods:**

In this retrospective study, 55 eyes of 54 consecutive patients were enrolled. Best-corrected visual acuity (BCVA) and spectral-domain optical coherence tomography (SD-OCT, Heidelberg, Spectralis) were performed preoperatively as well as 1, 3, 6, 9, and 12 months postoperatively. Special focus was put on the reintegration of outer retinal layers and the different ILM flap appearances.

**Results:**

FTMH closure rate was 100% (55/55). BCVA significantly improved over the follow-up period of 12 months from 0.98 ± 0.38 LogMAR preoperatively to 0.42 ± 0.33 LogMAR at 12 months postoperatively (*p* < 0.001). There was no significant correlation between the three different ILM flap appearances and BCVA. Better preoperative BCVA, complete restoration of the external limiting membrane (ELM), higher macular hole index (MHI), and smaller MH base diameter were associated with higher improvement of BCVA.

**Conclusion:**

Our study highlights the favorable morphological and functional outcomes of the I-ILM flap technique in the short as well as in the long term. While complete ELM restoration revealed to be an important factor for improvement in BCVA, the different postoperative ILM flap appearances seem not to be related to BCVA.

**Supplementary Information:**

The online version contains supplementary material available at 10.1007/s00417-021-05082-7.

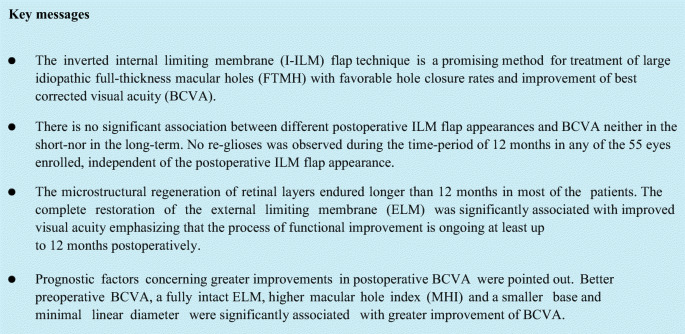



## Introduction

A full-thickness macular hole (FTMH) is a sight-threatening condition which is defined as a retinal defect arising from the internal limiting membrane (ILM) extending up to the retinal pigment epithelium (RPE) [1]. This vitreoretinal disorder is considered to be idiopathic in most of the cases and has an estimated incidence of 7.8 new cases per 100,000 population per year. It mostly occurs in individuals older than 65 years with a predominance of females [2–6].

In 1991, Kelly and Wendel first described pars plana vitrectomy (PPV) and fluid-gas exchange as an effective treatment of FTMH with macular hole (MH) closure in 58% and visual acuity (VA) improvement in 42% of all cases [7]. With the introduction of ILM peeling and the development of different staining agents for better visualization of the ILM, the success rate of MH surgery increased up to 97% [8–14]. Since then, PPV, ILM peeling, and fluid-gas exchange have become the standard surgical treatment for MH [15, 16].

However, in large MH (minimum linear diameter > 400 μm), anatomical and functional success is more difficult to achieve with reported closure rates between 50 and 88% [13, 17–21]. The risk of surgical failure is higher among large MH. Failure to close, reopening, flat-open closure, or flat MH margins with bare RPE occur more often than in MH with a minimum linear diameter < 400 μm [7, 21, 22]. Further effort in improving vitreoretinal surgery for MH led to the development of the inverted ILM (I-ILM) flap technique, first described by Michalewska et al. in 2010 [21]. This technique resulted in an improvement of closure rates and visual function in large MH. Since then, several studies have reported the superiority of the I-ILM flap technique over ILM peeling in the treatment of large MH achieving better anatomical and functional outcomes [22–28]. Further studies have demonstrated the dependence of functional improvement on the postoperative integrity of the outer retinal layers. Especially, the restoration of the external limiting membrane (ELM), ellipsoid zone (EZ), and outer photoreceptor segments (OS) were reported to significantly correlate with an improved postoperative best-corrected visual acuity (BCVA) [29–35]. Over the last 10 years, several modifications of the original I-ILM flap technique with similar favorable results have been described [36–42].

The development of intraoperative spectral-domain optical coherence tomography (iSD-OCT; Spectralis HRA Oct; Heidelberg Engineering, Heidelberg, Germany) further improved MH surgery, providing real-time information about the vitreous body, retinal layers, and its manipulation during vitreoretinal surgery. iSD-OCT proved to be an efficient tool in performing the I-ILM flap technique allowing a controlled ILM peeling and proper positioning of the ILM flap [43–47]. However, despite promising functional and anatomical results for large MH, some vitreoretinal surgeons are still reserved in performing this new technique because of concerns regarding the long-term outcomes. Especially, the microstructural regeneration of the outer retinal layers is a matter of concern [48]. The purpose of this study was to report the long-term anatomical and functional outcomes of the I-ILM flap technique in the treatment of large idiopathic FTMH. In regard to the anatomical outcomes, we put special focus on different postoperative ILM flap appearances and microstructural regeneration of the retinal layers using SD-OCT.

## Methods

### Study design

We retrospectively reviewed the medical records of all patients who underwent surgery for MH repair using the I-ILM flap technique at the University Hospital rechts der Isar of the Technical University Munich, Germany, between December 2009 and July 2020. Only patients with idiopathic FTMH in stage 3 or 4 according to Gass [49] and a minimum linear diameter of 400 μm were included. Exclusion criteria were coexisting ocular pathologies such as retinal vascular diseases (e.g., diabetic retinopathy, retinal vascular occlusion), age-related macular degeneration, glaucoma, history of previous retinal surgery, history of trauma, uveitis, high myopia (refractive error of more than − 6.00 diopters), or retinal detachment. Finally, 55 eyes of 54 consecutive patients were enrolled in this study. Prior to surgery, each patient was informed about the risks and benefits, and written informed consent was obtained from all patients. This study was approved by the local ethics committee of the Technical University Munich and adhered to the tenets of the Declaration of Helsinki.

All patients underwent a comprehensive ophthalmologic examination, including measurement of BCVA (decimal values) using the 4-m Snellen chart, slit-lamp biomicroscopy, intraocular pressure measurements, indirect ophthalmoscopy, and SD-OCT, at baseline as well as 1, 3, 6, 9, and 12 months postoperatively.

Further patient demographics collected included age, sex, duration of symptoms, lens status, and presence of an epiretinal membrane (ERM).

**Fig. 1:**
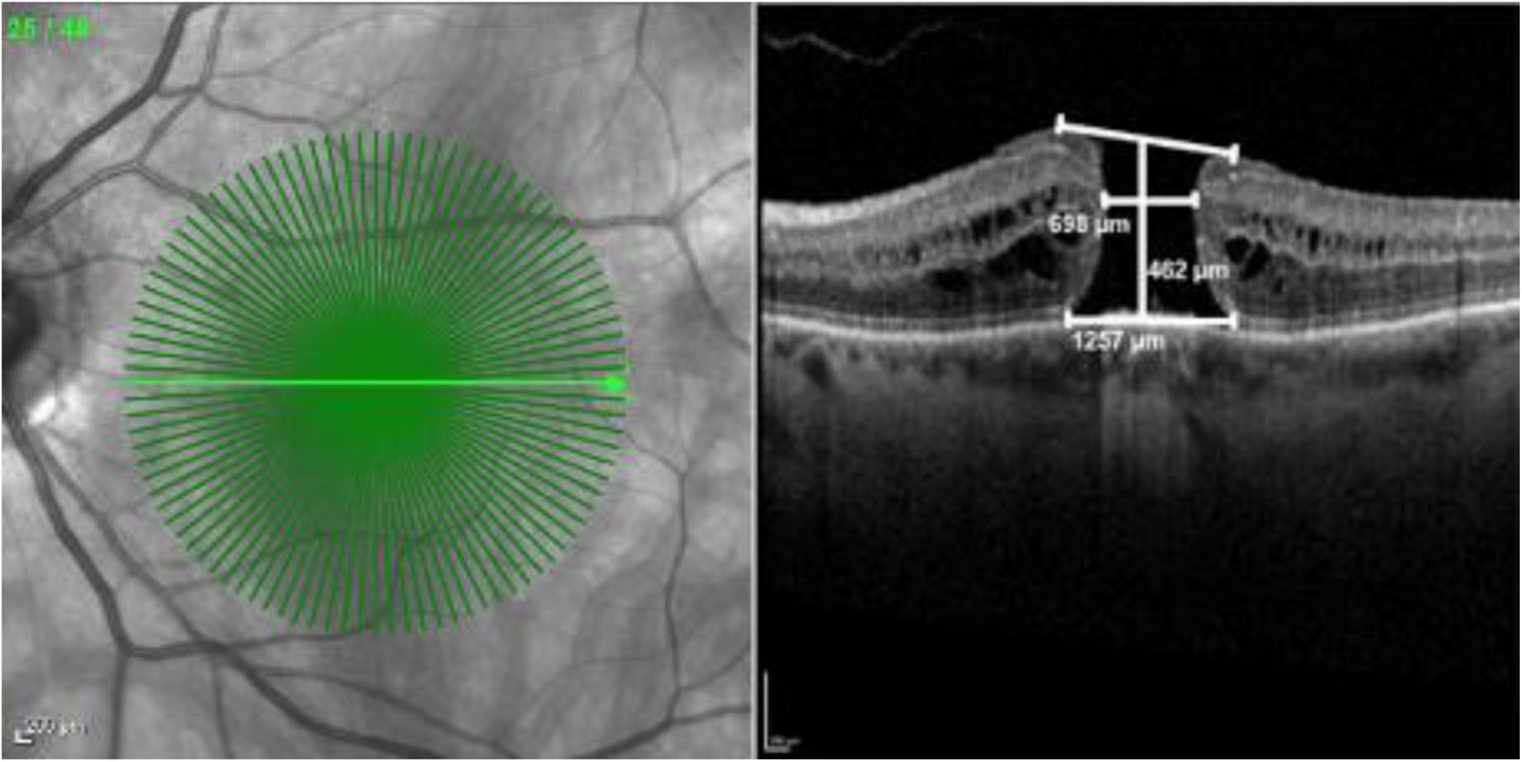
SD-OCT scan of a full-thickness macular hole (FTMH) using our measurement protocol with the caliper tool (Spectralis, Heidelberg). Minimum linear diameter 698 μm, base diameter 1257 μm, central retinal thickness 462 μm

**Fig. 2:**
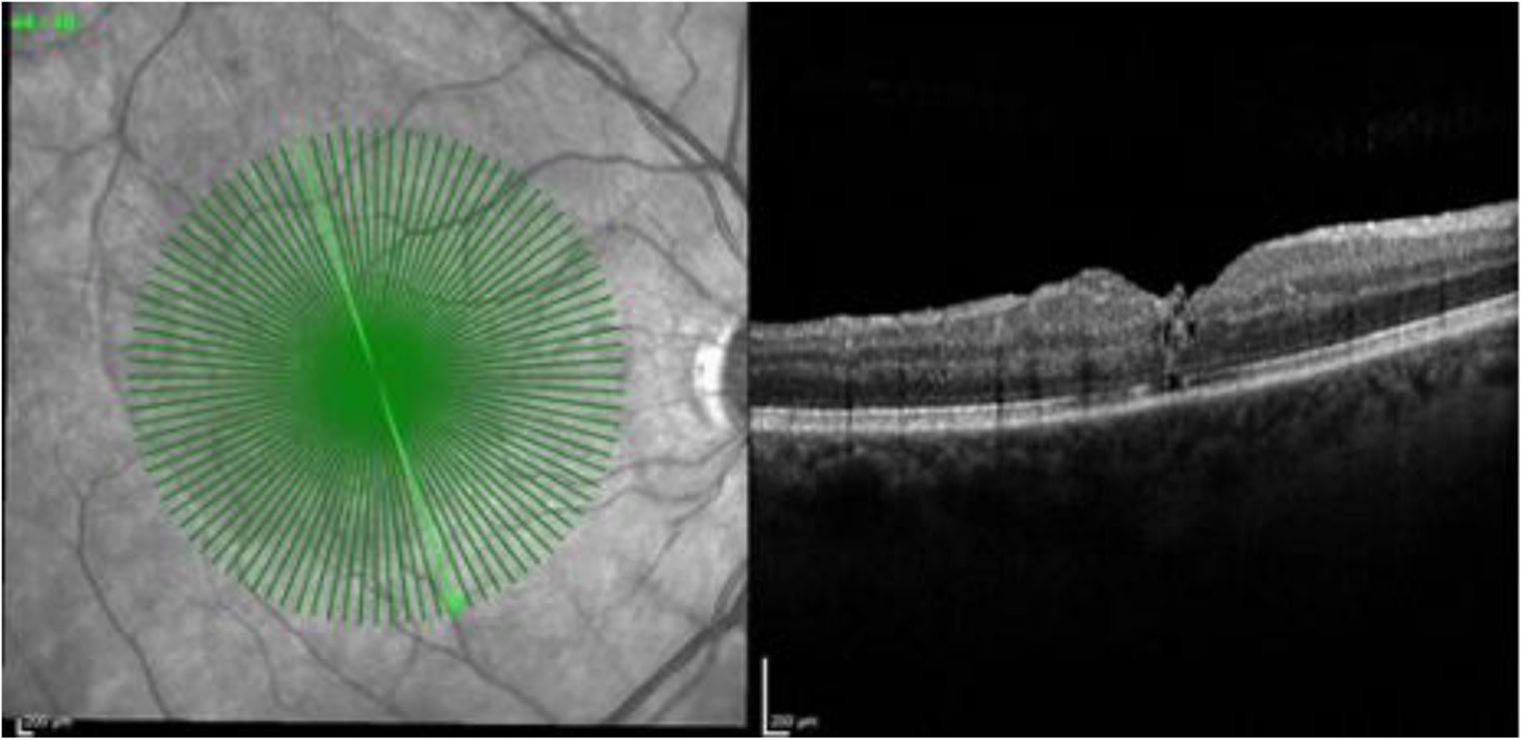
Optical coherence tomography scan of a closed FTMH at 3 months postoperative with gradings of ELM = 0 (continuous), EZ = 1 (disrupted), and OS = 0 (disrupted). The integrity of the ELM, EZ, and OS was nominally graded as 0 if the layer was fully restored and continuous or as 1 if the layer was absent or partially restored but disrupted

Pre- and postoperative SD-OCT measurements were taken using the same sectional images. The minimum linear and base diameters of the MH, the foveal configuration, and the central retinal thickness were assessed by SD-OCT. The minimum linear diameter (minimal extent of the MH parallel to the RPE), the base diameter (diameter at the level of the RPE), and the central retinal height (maximal distance between the RPE and the vitreoretinal interface) were measured using the caliper software tool. Our measurement protocol is visualized in Fig. 1. The macular hole index (MHI, ratio of the macular hole height to its base diameter) was calculated for each patient [50]. The layers were considered intact if a regular and continuous hyperreflective line corresponding to the EZ, ELM, or OS displayed in SD-OCT. Disrupted layers were characterized by hyporeflective discontinuities in the EZ, ELM, or OS line (Fig. 2). These classifications are based on the agreement of two authors (M.M. and N.B.). SD-OCT was used to confirm MH closure, defined by the integrity of the retinal layers in the macular area without evidence of a bare RPE. Flat-open and elevated-open closure-type configurations were considered as surgical failure. The primary outcome measure was defined as MH closure; secondary outcomes were postoperative BCVA, postoperative flap appearance, and the integrity of retinal layers.

### Surgical procedure

**Fig. 3:**
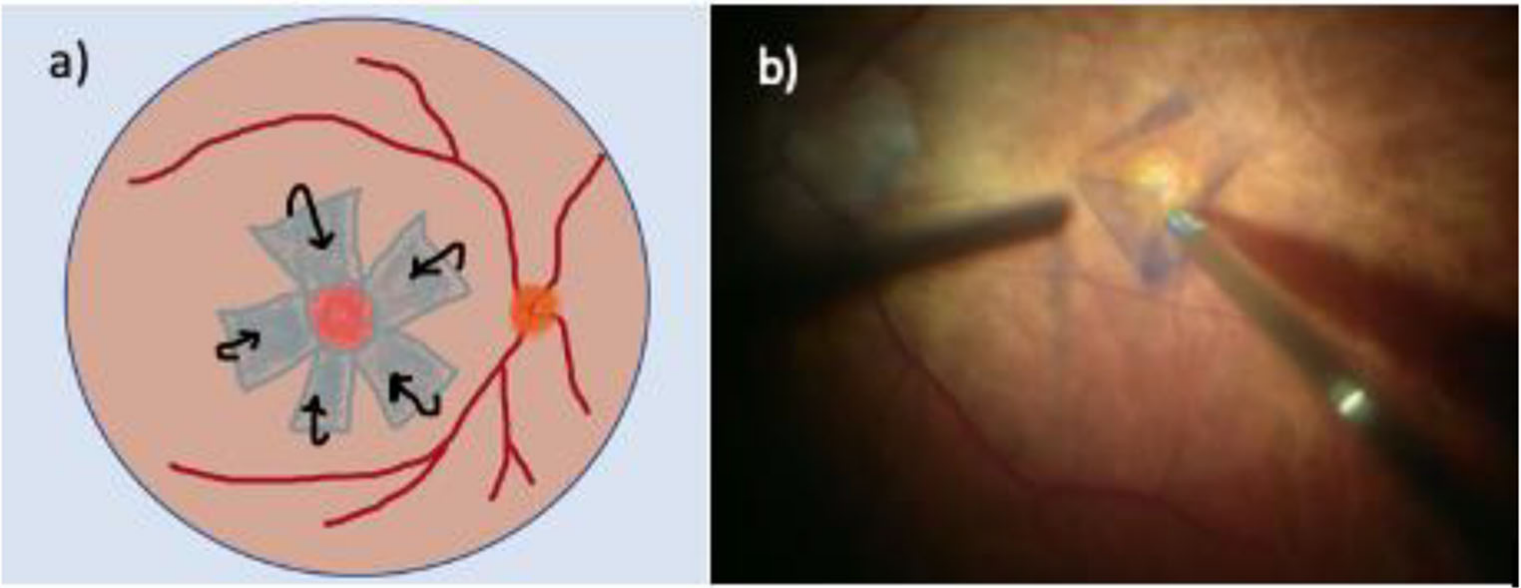
Inverted ILM flap technique. **a** Visualization of the inverted internal limiting membrane (I-ILM) flap technique in a schematic drawing. Circumferential ILM peeling 2.0–2.5 disk diameters surrounding the full-thickness macular hole (MH), leaving the edges attached to the margins of the FTMH. An ILM rosette is created and inverted to cover the FTMH. **b** Microscope image demonstrating the preparation of the ILM flap in a patient with large FTMH. Edges of the flap were trimmed with a vitreous cutter

Standard three-port 23-gauge pars plana vitrectomy (Constellation; Alcon Laboratories, Fort Worth, Texas, USA) with I-ILM flap technique was performed in all patients by one experienced vitreoretinal surgeon (M.M.). The vitrectomy was combined with phacoemulsification and intraocular lens implantation if a visually significant cataract was present. After core and peripheral vitrectomy, the ILM was stained with 0.025% Brilliant Blue G (Brilliant Peel, Fluoron, Germany) for about 30 s followed by the air-fluid exchange to wash the excess dye. A present ERM was differentiated from the ILM by its staining pattern and peeled consequently. Thereafter, the ILM was grasped and peeled off in a circumferential pattern for about 2.0–2.5 disk diameters surrounding the MH using end-gripping forceps, leaving the innermost part attached at the rim of the MH (Fig. 3). During this maneuver, perfusion was set at a low level. Edges of the ILM were trimmed with a vitreous cutter and the remnant was gently inverted to cover the MH in such a way that the surface which normally faced the vitreous body was now directed towards the RPE, covering the entire area of exposed RPE. No additional manipulation of the ILM flap was done hereafter. In all patients, fluid-air exchange and gas insufflation of 12% perfluoropropane (C3F8; Perfluoron, Alcon Laboratories, Fort Worth, Texas, USA) into the vitreous cavity was performed at the end of surgery (online resource 1). For dynamic intraoperative imaging, the microscope-integrated iSD-OCT system Rescan 700 (Carl Zeiss Meditec AG, Oberkochen, Germany) was used. The patients were advised to maintain a face-down position for 3 days postoperatively [46]. The I-ILM flap technique used in our study differed from the original I-ILM flap technique. Instead of creating a single I-ILM flap, a radial I-ILM flap, resembling a rosette, was peeled and inverted over the MH [21, 46].

Online resource 1This video shows the performance of the inverted internal limiting membrane (I-ILM) flap technique. After a standard three-port 23-gauge pars plana vitrectomy, the ILM was stained with 0.025% Brilliant Blue G followed by air-fluid exchange. Subsequently, the ILM was peeled off in a circular pattern for about 2.0-2.5 disk diameters surrounding the macular hole (MH) leaving the innermost part attached at the rim of the MH. The ILM remnants were gently inverted to fully cover the MH. Thereafter, fluid-air exchange and gas insufflation of 12% perfluoropropane into the vitreous cavity was performed (MOV 60060 kb)

### Statistical analysis

Numerical data was reported as mean ± standard deviation (SD) or median and range, whereas qualitative variables were presented as frequencies (absolutes) and percentages (%). For comparison of variables between two or more groups, the Mann-Whitney *U* test, the *t* test, or the chi-square test were used depending on the characteristics of the groups and the variables of the *t* test. The correlation between two continuous, non-normally distributed data were calculated with the Spearman correlation, the relationship between two continuous, normally distributed variables with the Pearson correlation. Paired *t* tests were conducted to evaluate postoperative changes in measured outcomes, with BCVA converted to the logarithm of the minimum angle of resolution (LogMAR) for analysis. Univariate variance models were conducted with postoperative BCVA as the dependent variable. Covariates and independent factors involved preoperative BCVA, age, gender, lens status, duration of symptoms, MH minimal diameter, MH base diameter, postoperative state of ELM, EZ, and OS as well as the postoperative appearance of an ILM flap. Factors which tested significantly in a univariate association were included in the multiple regression analysis to determine the factors significantly associated with postoperative BCVA. Statistical testing was performed at the 2-tailed alpha level of 0.05. All analyses were performed using SPSS (version 24.0; SPSS Inc., Chicago, Illinois, USA).

## Results

**Fig. 4:**
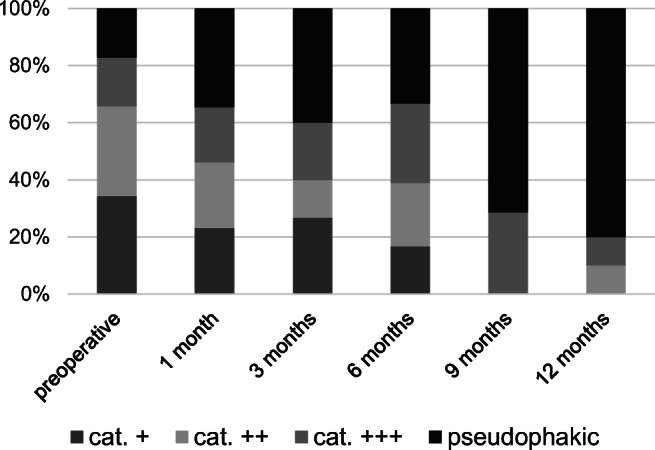
Patients’ lens status preoperatively as well as 1, 3, 6, 9, and 12 months postoperative. At 12 months postoperative, 80% of the patients were pseudophakic compared to 25.5% at baseline

A total of 55 eyes in 54 consecutive patients, 39 (70.9%) women and 16 (29.1%) men, with a mean age of 67 ± 7 years were included. At baseline, 41 (74.5%) patients were phakic and 14 (25.5%) patients were pseudophakic. Phacoemulsification and intraocular lens implantation were performed in combination with PPV in 13 cases (23.6%). The lens status of patients during the follow-up period is illustrated in Fig. 4. Lens status was included as a possible confounder variable in our statistical analysis.

On a SD-OCT examination at baseline, the mean minimal MH diameter was 517 μm (± 117 μm, range 401–863 μm), the mean MH base diameter was 1014 μm (± 368 μm, range 526–2389 μm), and the mean central retinal height was 413 μm (± 88 μm, range 243–758 μm). This results in a mean MHI of 0.45 ± 0.16. The MHI showed a significant negative correlation to BCVA (LogMAR) at 1, 3, 6, and 12 months postoperatively (*r* = − .35, *r* = − .42, *r* = − .48, *r* = − .67, *p* < 0.05). A higher MHI was associated with a lower LogMAR value in terms of a better VA. Patients’ demographics and baseline characteristics are reported in Table 1.

**Table 1:** Baseline characteristics of the patients (*n* = 54) and affected eyes (*n* = 55)

Age, years (mean ± SD; range)	67.0 (± 7.0)
Female gender, *n* (%)	39 (70.9%)
Right eye, *n* (%)	25 (45.5%)
Lens status, phakic	41 (74.5%)
ERM, *n* (%)	22 (40.0%)
Mean duration of symptoms, months (median)	7.3, range 0.5–28
Mean MH minimal linear diameter (μm)	517 (± 117), range 401–863
Mean MH base diameter (μm)	1014 (± 368), range 526–2389
Preoperative BCVA (mean LogMAR ± SD), Snellen	0.98 (± 0.38), 20/200

*IOL*, intraocular lens; *ERM*, epiretinal membrane; *BCVA*, best-corrected visual acuity; *logMAR*, logarithm of minimal angle of resolution; *MH*, macular hole; *SD*, standard deviation

**Fig. 5:**
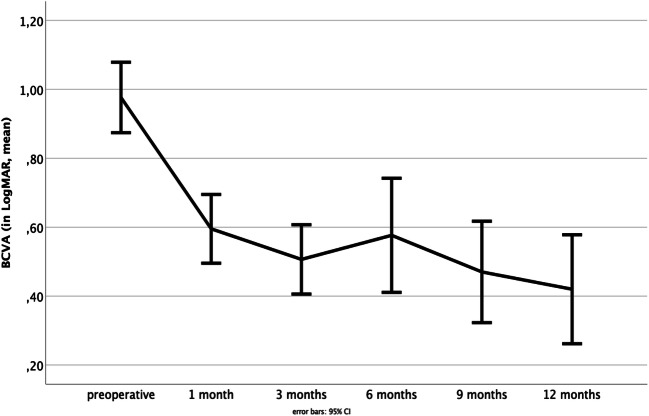
The mean BCVA improved from 0.98 ± 0.38 LogMAR preoperatively to 0.60 ± 0.34 to 0.51 ± 0.27, 0.58 ± 0.39, 0.47 ± 0.33, and 0.42 ± 0.33 at 1, 3, 6, 9, and 12 months after surgery, respectively (*p* < 0.001). Error bars 95% CI

The mean BCVA improved from 0.98 ± 0.38 LogMAR (Snellen’s equivalent 20/200) preoperatively to 0.60 ± 0.34 (Snellen’s equivalent 20/80), 0.51 ± 0.27 (Snellen’s equivalent 20/63), 0.58 ± 0.39 (Snellen’s equivalent 20/80), 0.47 ± 0.33 (Snellen’s equivalent 20/63), and 0.42 ± 0.33 LogMAR (Snellen’s equivalent 20/50) at 1, 3, 6, 9, and 12 months after surgery, respectively (*p* < 0.001). Changes in preoperative and postoperative BCVA are shown in Fig. 5. The mean improvement of BCVA equaled an improvement of 4 Snellen lines. There was no loss of BCVA in any of the patients.

Patients’ age significantly correlated with preoperative BCVA (*r* = .35, *p* < 0.05) but not with postoperative BCVA improvements. Regarding MH parameter, we found a significant correlation between the base diameter as well as the minimal linear diameter and BCVA (*r* = .83, *r* = .40, *p* < 0.01) as a larger base diameter and a larger minimal linear diameter were connected to higher values of LogMAR and thus poorer BCVA. Furthermore, final BCVA was associated with initial BCVA, as a worse preoperative BCVA came along with worse postoperative BCVA (*r* = .46, *p* < 0.01). No significant correlations were observed between gender, duration of symptoms, and improvements of BCVA in our study (*p* > 0.05).

The median duration of symptoms reported by the patients was 7.3 months (range 0.5 to 28 months). Interestingly, we found a statistically significant correlation between the duration of symptoms and the MH minimal diameter. A longer duration of symptoms was associated with a larger MH minimal diameter (*r* = .46, *p* < 0.01). Postoperative SD-OCT scans confirmed MH closure in 55 of 55 eyes within 3 months (closure rate 100%). No case of flat-open or elevated-open MH was found. No adverse events were recorded during surgery or the follow-up period of 12 months. Postoperative data are presented in Table 2.

**Table 2:** Postoperative characteristics

MH closure, *n* (%)	55 (100%)
BCVA 1 month postoperative (mean LogMAR ± SD), Snellen	0.60 (± 0.34), 20/80
BCVA 3 months postoperative (mean LogMAR ± SD), Snellen	0.51 (± 0.27), 20/63
BCVA 6 months postoperative (mean LogMAR ± SD), Snellen	0.58 (± 0.39), 20/80
BCVA 9 months postoperative (mean LogMAR ± SD), Snellen	0.47 (± 0.33), 20/63
BCVA 12 months postoperative (mean LogMAR ± SD), Snellen	0.42 (± 0.33) (20/50)

*BCVA*, best-corrected visual acuity; *logMAR*, logarithm of minimal angle of resolution; *MH*, macular hole; *SD*, standard deviation

**Fig. 6:**
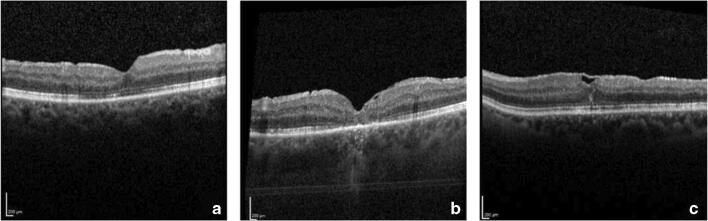
The three different ILM flap appearances. In type A, the ILM flap was not visible at all (32.1%, *n* = 17); in type B, the ILM flap was partly visible (18.9%, *n* = 10); and in type C, the ILM flap was visible over the whole foveal area (49.1%, *n* = 26)

SD-OCT enabled us to distinguish between three different postoperative ILM flap appearances (Fig. 6). In type A, the ILM flap was not visible at all (32.1%, *n* = 17); in type B, the ILM flap was partly visible (18.9%, *n* = 10); and in type C, the ILM flap was visible over the whole foveal area (49.1%, *n* = 26). Statistical analysis did not show a significant association between the different postoperative ILM flap appearances and BCVA (*p* > 0.05). The ILM flap appearance was categorized at each follow-up time point. Remarkably, the ILM flap appearance type remained stable for each eye over the follow-up period.

An ERM was detected and peeled in 22 (40.0%) eyes. We did not detect an excessive proliferation of glia cells in terms of gliosis in SD-OCT during the 12-month follow-up period in any eye.

**Fig. 7:**
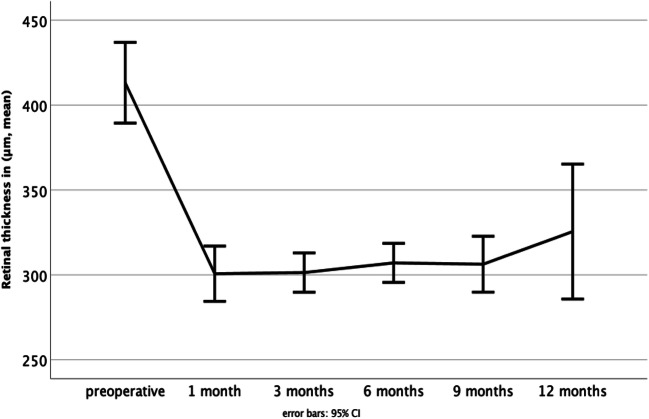
The mean retinal thickness (in μm) 1 month postoperative decreased significantly compared to the preoperative mean retinal thickness (*p* > 0.001). There were no significant differences in retinal thickness over further time points. Error bars 95% CI

There was a significant reduction of retinal thickness 1 month postoperatively compared to baseline retinal thickness (*p* > 0.001). During the further follow-up period, retinal thickness stayed constant (Fig. 7). Retinal thickness and BCVA did not correlate significantly (*p* > 0.05).

The retrospective design of our study led to missing data at different examination time points. Although a complete assessment after 1, 3, 6, 9, and 12 months postoperatively is the scheduled standard procedure in our clinic, only a few patients met each appointment. However, each patient attended two or more examinations. Additionally, the examination intervals differed because of organizational limitations. The exact follow-up characteristics are illustrated in Table 3.

**Table 3:** Follow-up characteristics of the patients (*n* = 54) and affected eyes (*n* = 55)

Follow-up time point	Eyes (*n*, %)	Examination interval in months (mean ± SD, range)
1 month postoperatively	46 (83.6%)	1.28 ± 0.4 (0.6–2.1)
3 months postoperatively	30 (54.5%)	3.25 ± 0.7 (2.2–4.6)
6 months postoperatively	22 (40%)	5.95 ± 0.8 (4.5–7.1)
9 months postoperatively	20 (36.4%)	9.21 ± 1.0 (7.9–11.4)
12 months postoperatively	17 (30.9%)	14.3 ± 5.1 (11.4–34.3)

*SD*, standard deviation

**Fig. 8:**
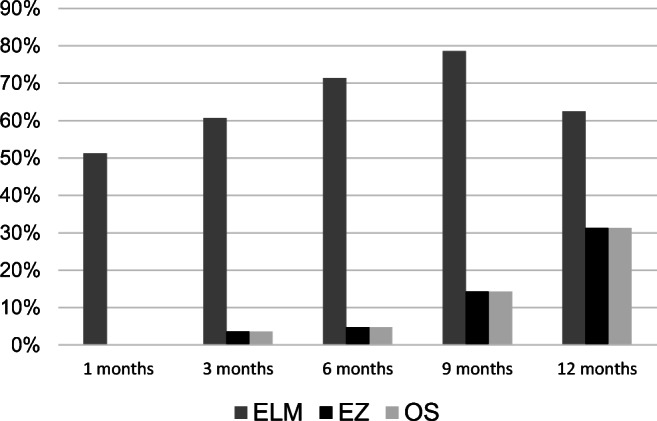
Regeneration of retinal layers in percentage after 1, 3, 6, 9, and 12 months postoperative. SD-OCT showed complete restoration of foveal microstructures (ELM, EZ, and OS) at the end of the follow-up period in 31.3% (5/16) of the eyes. The ELM, EZ, and OS were fully restored in 62.5% (10/16), 31.3% (5/16), and 31.3% (5/16) eyes at the end of the follow-up period, respectively. Restoration of the ELM preceded the restoration of the EZ and OS in all cases

Only 17 eyes (30.9%) completed the whole examination period. In one of these patients, SD-OCT was not evaluated due to poor quality. In the remaining patients, SD-OCT showed complete restoration of foveal microstructures (ELM, EZ, and OS) at the end of the follow-up period in 31.3% (5/16) of the eyes. The ELM, EZ, and OS were fully restored in 62.5% (10/16), 31.3% (5/16), and 31.3% (5/16) eyes at the end of the follow-up period, respectively. Postoperative recovery of the photoreceptor layers is illustrated in Fig. 8. Complete EZ and OS restoration was not observed without complete restoration of the ELM. Restoration of the ELM preceded the restoration of the EZ and OS in all cases.

Univariate variance analysis was conducted to detect differences between BCVA of patients with a complete restoration of ELM, EZ, and OS compared to patients with ongoing defects regarding the foveal microstructure. ELM defects were associated with poorer BCVA compared to patients with an intact ELM after 1, 3, 6, 9, and 12 months postoperatively (*p* < 0.05). In our study, EZ and OS were either both intact or both impaired (*r* = 1.00, *p* < 0.001). Therefore, we studied them as one factor in a univariate variance analysis. There was a significant association between ongoing EZ and OS defects and poorer BCVA after 1 month postoperatively (*p* < 0.01).

A stepwise multiple regression analysis was conducted to predict improvement of BCVA after 12 months postoperatively based on the minimal linear diameter, MHI, and preoperative BCVA. A significant regression equation was found (*F*(3.13) = 13.9, *p* < 0.001), with an *R*^2^ of .76. Preoperative BCVA and MHI were included in the explanatory model since they were identified to be significant predictors of improvement in BCVA after 12 months (*p* = 0.001, *p* > 0.001, respectively, Table 4).

**Table 4:** Independent factors associated with the change of BCVA after 12 months by multiple linear regression with a stepwise approach, *R*^2^ = 0.76

Multiple regression analysis	Standardized coefficient	*p* value
Preoperative BCVA (LogMAR)	− 0.85	0.000
MHI	− 0.74	0.001

*BCVA*, best-corrected visual acuity; *logMAR*, logarithm of minimal angle of resolution; *MHI*, macular hole index

**Fig. 9:**
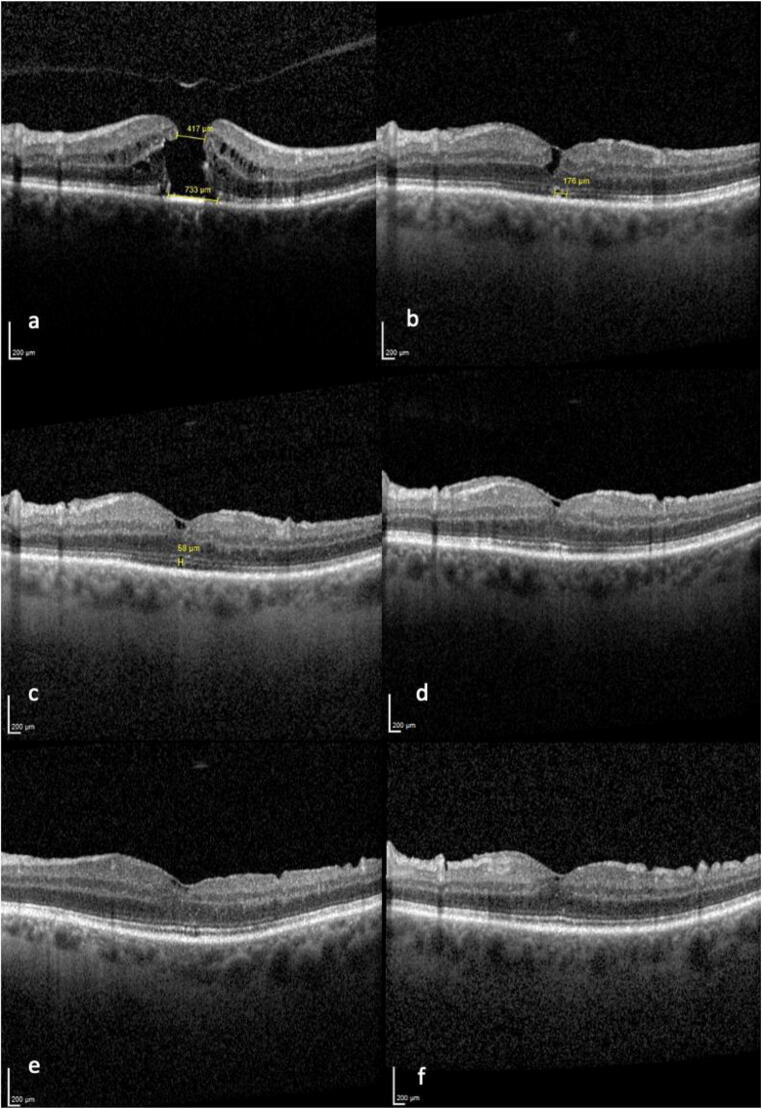
Representative case of a 70-year-old patient with large FTMH (417 μm), preoperatively (**a**); 3 months postoperatively, ELM intact, EZ/OS defects (**b**); 6 months postoperatively, ELM intact, EZ/OS defects (**c**); 9 months postoperatively, ELM and EZ/OS intact (**d**), 12 months postoperatively, ELM and EZ/OS intact (**e**). BCVA (decimal) improved from 0.1 preoperatively to 0.3 after 3 and 6 months and to 0.6 after 9 and 12 months, respectively. An additional follow-up measurement after 24 months showed a stable visual acuity of 0.6, ELM and EZ/OS intact (**f**)

iSD-OCT imaging allowed real-time visualization of the retinal structures and its manipulation with vitreoretinal instruments during the whole surgery. Secure and controlled ILM peeling as well as the positioning of the I-ILM flap was successfully performed in all eyes. At the end of the surgery, a complete fluid-air exchange and a proper position of the ILM flap covering the MH was confirmed with iSD-OCT in all cases. Figure 9 shows a representative case demonstrating SD-OCT imaging during the follow-up period of 12 months.

## Discussion

In 2010, Michalewska et al. proposed an approach for the initial surgery of large MH, in which the ILM is not completely removed, but a small remnant is left attached to the margins of the MH to cover it [21]. Although PPV, ILM peeling, and fluid-gas exchange still remain the standard surgical treatment for MH, several studies showed the superiority of the I-ILM flap technique. Especially, for large MH (diameter > 400 μm), higher closure rates and improvements in BCVA have been reported [26–28, 35, 36, 51]. A meta-analysis of Gu and Qiu concluded that the I-ILM flap technique is an effective and safe method for the treatment of large MH, with high closure rates and good functional outcomes [26]. Shen et al. conducted a meta-analysis to compare the efficacy of the I-ILM flap technique and ILM peeling for treating large MH. Their data demonstrated a statistically significant higher closure rate in MH treated with I-ILM flap technique than with ILM peeling. After 3 months postoperatively, BCVA was significantly better with the I-ILM flap technique compared to ILM peeling only. However, there was no difference in visual outcomes between the two groups at the follow-up measurement 6 months postoperatively [28].

As stated in the “Introduction” section, our main objective was to analyze the functional and morphological outcomes of the I-ILM flap technique during a time period of 12 months. Our study emphasizes the good anatomic and functional results of the I-ILM flap technique in the short as well as in the long term. Compared to preoperative BCVA, postoperative BCVA improved statistically significant over all measurement time points and the MH closure rate was 100%.

The closure mechanism of MH treated with the I-ILM flap technique is yet not fully understood. The ILM flap seems to work as a scaffold for the proliferation and migration of Müller cells [26]. Migrated Müller cells as well as the ILM flap itself provide neurotrophic factors and basic fibroblast growth factor (bFGF) which induce glia cell proliferation, possibly leading to MH closure [26, 48, 52, 53]. Shiode et al. identified histological components which increase the proliferation of Müller cells (type 4 collagen, fibronectin, laminin). Eventually, it may be possible to use them as intraoperative adjuvants to fasten up hole closure in the future [46, 52]. Further studies are pivotal to understand the MH closure mechanism and to investigate the effects of type IV collagen, fibronectin, and laminin on MH closure [52].

Several studies found an association between the microstructural regeneration of retinal layers and the improvement of BCVA [29, 34, 46, 51, 54–56]. Especially, the complete reintegration of the ELM was identified to be a prognostic factor for the improvement of BCVA [30, 34, 46, 55, 57, 58]. The microstructural regeneration of retinal layers can endure up to 2 years. In this context, it is important to educate the patients about the ongoing process of functional improvement [48]. In our study, BCVA improved even after 12 months postoperatively. We also identified the integrity of ELM correlating with an improvement of BCVA. Consistent with findings in other studies, the reintegration of ELM always preceded the reintegration of the EZ and OS in our study [29, 53, 54, 59–61]. The I-ILM flap technique helps to restore foveal architecture [36, 51].

The development of SD-OCT facilitated analysis of morphological retinal aspects and their correlation with functional and anatomical results. Various authors studied prognostic factors in terms of morphological aspects and functional outcome, observing a correlation between MH size, MH base diameter, and improvement of BCVA [1, 18, 22, 59]. In our study, some prognostic factors could be identified. We found statistically significant correlations between the base diameter of MH and improvement of BCVA. A large MH basis correlated with a poorer BCVA in our study. Kusuhara et al. as well as Ruiz-Moreno et al. found significant correlations between the MHI and postoperative BCVA [50]. In our study, a greater improvement of BCVA was observed in patients with higher MHI values, confirming the observations of the formerly mentioned authors. Interestingly, this correlation was especially strong concerning BCVA values after 12 months. This indicates the MHI to be a good prognostic factor in the long term.

According to Kazmerczak et al., a short duration of symptoms was associated with higher improvement of BCVA [54]. We did not observe this correlation. Remarkably, a longer duration of symptoms was associated with larger MH minimal diameter in our study. Therefore, fast diagnosis and intervention seem to be very important to guarantee the best possible outcome.

Boniska et al. observed hyperreflective remnants of the ILM on the retinal surface in eyes operated with the I-ILM flap technique. It did not change over time and had no influence on the final VA [59]. To the best of our knowledge, we are the first to describe three different ILM flap appearances following the I-ILM flap technique [46]. In this study, we analyzed those different groups in terms of BCVA and re-gliosis. We did not find any correlation between different ILM flap appearance and BCVA. The reason for the distinct ILM flap appearance remains unclear and requires further histological studies. Gliosis induced by the proliferation of Müller cells is effective in closing MH, though excessive gliosis has cytotoxic effects on retinal neurons and may lead to worse BCVA [52]. Liu et al. reported hyperreflective foveal lesions in SD-OCT as a possible indicator for excessive foveal glia cell proliferation. An aberrant active glial proliferation in the macular area is associated with worse VA [61]. However, in our study, there was no indication for re-gliosis in any patient in the long-term cohort. One explanation for this finding could be the use of the “cover” technique instead of the “filling” technique [37].

Some concerns with the I-ILM flap technique have been evoked. The difficulty of the surgical procedure implicates a steep learning curve as the ILM only needs to be removed partly leaving their remnants attached to the margins of the MH. One main concern is the occurrence of ILM flap displacement, especially during fluid-air exchange [21]. Further complications associated with this technique could be damage to Müller cells, the formation of paracentral retinal holes, retinal thinning, and RPE atrophy [38, 54, 60, 62]. In our study, we did not find any of these complications. Particularly, we monitored the retinal thickness and did not find a statistically significant retinal thinning over 12 months. In order to encounter some of the risks, we used iSD-OCT when performing the I-ILM flap technique. iSD-OCT is a useful tool that provides valuable real-time information during surgery allowing to individualize surgical treatment for each patient [63]. The usage of iSD-OCT allows a safe and controlled performance of the I-ILM flap technique [11, 17, 44–47]. iSD-OCT helped us to confirm the correct positioning of the ILM flap at the very end of surgery. Furthermore, we controlled for a dry milieu after a fluid-air exchange, which is very important for hole closure [52].

Over the last years, several modifications of the original I-ILM flap technique have been developed, e.g., variations of the size, shape, number, and manner in which the flaps are put on the MHs as well as the usage of perfluoro-n-octane (PFO), different dyes, autologous blood, and adhesive viscoelastics [36–42]. These various modifications with their concomitant risks and benefits need to be further explored. In our study, the I-ILM flap technique also was performed in a modified version as a radial I-ILM flap (I-ILM flap rosette) was used to cover the MH instead of a single I-ILM flap [46].

There are a few limitations to our study. First of all, the retrospective character of our study led to variations of measurement time points and missing data during the follow-up (Table 3). The statistical power is reduced due to the missing data and our results need to be interpretated cautiously. Further studies with a larger sample size should validate these results. Secondly, the subgroups concerning the different ILM flap appearances were relatively small which limited the statistical strength. Further long-term studies with bigger subgroups should address these different ILM flap appearances and their possible effects on BCVA and re-gliosis. The strength of our study lies in the long follow-up period over 12 months and the standardized surgery procedure which was conducted by one surgeon (M.M.) in all cases. Future studies should examine even longer follow-up periods to investigate if changes in BCVA are ongoing over 12 months.

## Conclusion

The findings of this study indicate the I-ILM flap technique to be an effective and safe method for treating large idiopathic MH, with favorable short- as well as long-term results. In our study, each MH was closed at 3 months postoperatively and BCVA significantly improved. This work has highlighted a few prognostic factors concerning the postoperative development of BCVA. Better preoperative BCVA, complete restoration of the ELM, smaller MH base diameter, and higher MHI were significantly associated with greater improvement of postoperative BCVA. The results support the idea that the reintegration of outer retinal layers is an ongoing process leading to improvements of BCVA at least over 12 months. In this study, there was no association between different postoperative ILM flap appearances and BCVA in our study. Larger case series are required to further understand MH closure and long-term results. Further indications should be considered for applying the promising I-ILM flap technique, e.g., an unfavorable MH constitution (large base), AMD, or worse BCVA in the contralateral eye [46].

## Data Availability

All the data are available upon request.

## References

[1] Duker J, Kaiser P, Binder S, de Smet M, Gaudric A, Reichel E, Sadda S, Sebag J, Spaide R, Stalmans P (2013). The international vitreomacular traction study group classification of vitreomacular adhesion, traction, and macular hole. Ophthalmology.

[2] Wang S, Xu L, Jonas J (2006). Prevalence of full-thickness macular holes in urban and rural adult Chinese: the Beijing eye study. Am J Ophthalmol.

[3] Jackson T, Donachie P, Sparrow J, Johnston R (2013). United Kingdom National Ophthalmology Database Study of Vitreoretinal Surgery: Report 2, Macular Hole. Ophthalmology.

[4] McCannel C, Ensminger J, Diehl N, Hodge D (2009). Population-based incidence of macular holes. Ophthalmology.

[5] Ezra E (2001). Idiopathic full thickness macular hole: natural history and pathogenesis. Br J Ophthalmol.

[6] Steel D, Lotery A (2013). Idiopathic vitreomacular traction and macular hole: a comprehensive review of pathophysiology, diagnosis, and treatment. Eye.

[7] Kelly NE, Wendel RT (1991). Vitreous surgery for idiopathic macular holes: results of a pilot study. Arch Ophthalmol.

[8] Eckardt CL, Eckardt U, Groos ST, Luciano LI, Reale EN (1997). Removal of the internal limiting membrane in macular holes. Clinical and morphological findings. Ophthalmologe.

[9] Almony A, Nudleman E, Shah GK, Blinder KJ, Eliott DB, Mittra RA, Tewari A (2012). Techniques, rationale, and outcomes of internal limiting membrane peeling. Retina.

[10] Kadonosono K, Itoh N, Uchio E, Nakamura S, Ohno S (2000). Staining of internal limiting membrane in macular hole surgery. Arch Ophthalmol.

[11] Haritoglou C, Gandorfer A, Gass CA, Schaumberger M, Ulbig M, Kampik A (2002). Indocyanine green-assisted peeling of the internal limiting membrane in macular hole surgery affects visual outcome: a clinicopathologic correlation. Am J Ophthalmol.

[12] Beutel J, Dahmen G, Ziegler A, Hoerauf H (2007). Internal limiting membrane peeling with indocyanine green or trypan blue in macular hole surgery: a randomized trial. Arch Ophthalmol.

[13] Williamson TH, Lee E (2014). Idiopathic macular hole: analysis of visual outcomes and the use of indocyanine green or brilliant blue for internal limiting membrane peel. Graefes Arch Clin Exp Ophthalmol.

[14] Bae K, Kang SW, Kim JH, Kim SJ, Kim JM, Yoon JM (2016). Extent of internal limiting membrane peeling and its impact on macular hole surgery outcomes: a randomized trial. Am J Ophthalmol.

[15] Benson WE, Cruickshanks KC, Fong DS, Williams GA, Bloome MA, Frambach DA (2001). Surgical management of macular holes: a report by the American Academy of Ophthalmology. Ophthalmology.

[16] Parravano M, Giansanti F, Eandi CM, Yap YC, Rizzo S, Virgili G (2015). Vitrectomy for idiopathic macular hole. Cochrane Database Syst Rev.

[17] Ip MS, Baker BJ, Duker JS, Reichel E, Baumal CR, Gangnon R, Puliafito CA (2002). Anatomical outcomes of surgery for idiopathic macular hole as determined by optical coherence tomography. Arch Ophthalmol.

[18] Ullrich S, Haritoglou C, Gass C, Schaumberger M, Ulbig MW, Kampik A (2002). Macular hole size as a prognostic factor in macular hole surgery. Br J Ophthalmol.

[19] Salter AB, Folgar FA, Weissbrot J, Wald KJ (2012). Macular hole surgery prognostic success rates based on macular hole size. Ophthalmic Surg Lasers Imaging.

[20] García-Layana A, García-Arumí J, Ruiz-Moreno JS, Arias-Barquet L, Cabrera-López F, Figueroa MS (2015). A review of current management of vitreomacular traction and macular hole. J Ophthalmol.

[21] Michalewska Z, Michalewski J, Adelman RA, Nawrocki J (2010). Inverted internal limiting membrane flap technique for large macular holes. Ophthalmology.

[22] Yamashita T, Sakamoto T, Terasaki H, Iwasaki M, Ogushi Y, Okamoto F, Takeuchi M, Yasukawa T, Takamura Y, Ogata N, Nakamura Y, writing committee of Japan-Clinical Retina Research Team (J-CREST) (2018). Best surgical technique and outcomes for large macular holes: retrospective multicentre study in Japan. Acta Ophthalmol.

[23] Yuan J, Zhang LL, Lu YJ, Han MY, Yu AH, Cai XJ (2017). Vitrectomy with internal limiting membrane peeling versus inverted internal limiting membrane flap technique for macular hole-induced retinal detachment: a systematic review of literature and meta-analysis. BMC Ophthalmol.

[24] Kannan NB, Kohli P, Parida H, Adenuga OO, Ramasamy K (2018). Comparative study of inverted internal limiting membrane (ILM) flap and ILM peeling technique in large macular holes: a randomized-control trial. BMC Ophthalmol.

[25] Narayanan R, Singh SR, Taylor S, Berrocal MH, Chhablani J, Tyagi M, Ohno-Matsui K, Pappuru RR, Apte RS (2019). Surgical outcomes after inverted internal limiting membrane flap versus conventional peeling for very large macular holes. Retina.

[26] Gu C, Qiu Q (2018). Inverted internal limiting membrane flap technique for large macular holes: a systematic review and single-arm meta-analysis. Graefes Arch Clin Exp Ophthalmol.

[27] Rizzo S, Tartaro R, Barca F, Caporossi T, Bacherini D, Giansanti F (2018). Internal limiting membrane peeling versus inverted flap technique for treatment of full-thickness macular holes: a comparative study in a large series of patients. Retina.

[28] Shen Y, Lin X, Zhang L, Wu M (2020). Comparative efficacy evaluation of inverted internal limiting membrane flap technique and internal limiting membrane peeling in large macular holes: a systematic review and meta-analysis. BMC Ophthalmol.

[29] Itoh Y, Inoue M, Rii T, Hiraoka T, Hirakata A (2012). Significant correlation between visual acuity and recovery of foveal cone microstructures after macular hole surgery. Am J Ophthalmol.

[30] Hayashi H, Kuriyama S (2014). Foveal microstructure in macular holes surgically closed by inverted internal limiting membrane flap technique. Retina.

[31] Chang YC, Lin WN, Chen KJ, Wu HJ, Lee CL, Chen CH, Wu KY, Wu WC (2015). Correlation between the dynamic postoperative visual outcome and the restoration of foveal microstructures after macular hole surgery. Am J Ophthalmol.

[32] Iwasaki M, Kinoshita T, Miyamoto H, Imaizumi H (2019). Influence of inverted internal limiting membrane flap technique on the outer retinal layer structures after a large macular hole surgery. Retina.

[33] Faria M, Proença H, Ferreira N, Sousa D, Neto E, Marques-Neves C (2020). Inverted internal limiting membrane flap techniques and outer retinal layer structures. Retina.

[34] Baumann C, Kaye S, Iannetta D, Sultan Z, Dwivedi R, Pearce I (2019). Effect of inverted internal limiting membrane flap on closure rate, postoperative visual acuity, and restoration of outer retinal layers in primary idiopathic macular hole surgery. Retina.

[35] Ramtohul P, Parrat E, Denis D, Lorenzi U (2020). Inverted internal limiting membrane flap technique versus complete internal limiting membrane peeling in large macular hole surgery: a comparative study. BMC Ophthalmol.

[36] Velez-Montoya R, Ramirez-Estudillo J, Sjoholm-Gomez de Liano C, Bejar-Cornejo F, Sanchez-Ramos J, Guerrero-Naranjo J, Morales-Canton V, Hernandez-Da Mota S (2018). Inverted ILM flap, free ILM flap and conventional ILM peeling for large macular holes. Int J Retina Vitreous.

[37] Park J, Lee S, Park S, Lee J, Byon I (2018). Comparative analysis of large macular hole surgery using an internal limiting membrane insertion versus inverted flap technique. Br J Ophthalmol.

[38] Ghassemi F, Khojasteh H, Khodabande A, Dalvin L, Mazloumi M, Riazi-Esfahani H, Mirghorbani M (2019). Comparison of three different techniques of inverted internal limiting membrane flap in treatment of large idiopathic full-thickness macular hole. Clin Ophthalmol.

[39] Xu Q, Luan J (2020). Internal limiting membrane flap technique in macular hole surgery. Int J Ophthalmol.

[40] Michalewska Z, Michalewski J, Dulczewska-Cichecka K, Adelman R, Nawrocki J (2015). Temporal inverted internal limiting membrane flap technique versus classic inverted internal limiting membrane flap technique. Retina.

[41] Shin M, Park K, Park S, Byon I, Lee J (2014). Perfluoro-n-octane–assisted single-layered inverted internal limiting membrane flap technique for macular hole surgery. Retina.

[42] Casini G, Mura M, Figus M, Loiudice P, Peiretti E, De Cillà S, Fuentes T, Nasini F (2017). Inverted internal limiting membrane flap technique for macular hole surgery without extra manipulation of the flap. Retina.

[43] Riazi-Esfahani M, Khademi MR, Mazloumi M, Khodabandeh A, Riazi-Esfahani H (2015). Macular surgery using intraoperative spectral domain optical coherence tomography. J Ophthalmic Vis Res.

[44] Hattenbach L, Framme C, Junker B, Pielen A, Agostini H, Maier M (2016). Intraoperative Echtzeit-OCT in der Makulachirurgie [Intraoperative real-time OCT in macular surgery]. Ophthalmologe.

[45] Borrelli E, Palmieri M, Aharrh-Gnama A, Ciciarelli V, Mastropasqua R, Carpineto P (2018). Intraoperative optical coherence tomography in the full-thickness macular hole surgery with internal limiting membrane inverted flap placement. Int Ophthalmol.

[46] Maier M, Bohnacker S, Klein J, Klaas J, Feucht N, Nasseri A, Lohmann C (2018). Vitrektomie mit iOCT-assistierter invertierter ILM-Flap-Technik bei großen Makulaforamina. Ophthalmologe.

[47] Lytvynchuk LM, Falkner-Radler CI, Krepler K, Glittenberg C, Ahmed D, Petrovski G, Lorenz B, Ansari-Shahrezaei S, Binder S (2019). Dynamic intraoperative optical coherence tomography for inverted internal limiting membrane flap technique in large macular hole surgery. Graefes Arch Clin Exp Ophthalmol.

[48] Vieregge M, Valmaggia C, Scholl H, Guber J (2018). Microstructural retinal regeneration after internal limiting membrane flap surgery for repair of large macular holes: a 1-year follow-up study. Int Ophthalmol.

[49] Gass J (1988). Idiopathic senile macular hole. Its early stages and pathogenesis. Arch Ophthalmol.

[50] Kusuhara S, Teraoka Escaño M, Fujii S, Nakanishi Y, Tamura Y, Nagai A, Yamamoto H, Tsukahara Y, Negi A (2004). Prediction of postoperative visual outcome based on hole configuration by optical coherence tomography in eyes with idiopathic macular holes. Am J Ophthalmol.

[51] Hu X, Pan Q, Zheng J, Zhang Z (2019). Foveal microstructure and visual outcomes of myopic macular hole surgery with or without the inverted internal limiting membrane flap technique. Br J Ophthalmol.

[52] Shiode Y, Morizane Y, Matoba R, Hirano M, Doi S, Toshima S, Takahashi K, Araki R, Kanzaki Y, Hosogi M, Yonezawa T, Yoshida A, Shiraga F (2017). The role of inverted internal limiting membrane flap in macular hole closure. Invest Opthalmol Vis Sci.

[53] Oh J, Yang SM, Choi YM, Kim SW, Huh K (2012). Glial proliferation after vitrectomy for a macular hole: a spectral domain optical coherence tomography study. Graefes Arch Clin Exp Ophthalmol.

[54] Kaźmierczak K, Stafiej J, Stachura J, Żuchowski P, Malukiewicz G (2018). Long-term anatomic and functional outcomes after macular hole surgery. J Ophthalmol.

[55] Caprani S, Donati S, Bartalena L, Vinciguerra R, Mariotti C, Testa F, Porta G, Azzolini C (2017). Macular hole surgery: the healing process of outer retinal layers to visual acuity recovery. Eur J Ophthalmol.

[56] Villate N, Lee J, Venkatraman A, Smiddy W (2005). Photoreceptor layer features in eyes with closed macular holes: optical coherence tomography findings and correlation with visual outcomes. Am J Ophthalmol.

[57] Wakabayashi T, Fujiwara M, Sakaguchi H, Kusaka S, Oshima Y (2010). Foveal microstructure and visual acuity in surgically closed macular holes: spectral-domain optical coherence tomographic analysis. Ophthalmology.

[58] Shimozono M, Oishi A, Hata M, Kurimoto Y (2011). Restoration of the photoreceptor outer segment and visual outcomes after macular hole closure: spectral-domain optical coherence tomography analysis. Graefes Arch Clin Exp Ophthalmol.

[59] Bonińska K, Nawrocki J, Michalewska Z (2018). Mechanism of “flap closure” after the inverted internal limiting membrane flap technique. Retina.

[60] Takai Y, Tanito M, Sugihara K, Ohira A (2019). The role of single-layered flap in temporal inverted internal limiting membrane flap technique for macular holes: pros and cons. J Ophthalmol.

[61] Liu Y, Wu C, Wang Y, Dong Y, Liang D, Xiao B, Han Q, Chu Y (2019). Risk factors for glial cell proliferation after idiopathic macular hole repair with internal limiting membrane flap. BMC Ophthalmol.

[62] Imai H, Azumi A (2014). The expansion of RPE atrophy after the inverted ILM flap technique for a chronic large macular hole. Case Rep Ophthalmol.

[63] Hernan Gonzalez-Cortes J, Olvera-Barrios A, Emiliano Gonzalez-Cantu J, Mohamed-Hamsho J (2019) Microscope-integrated intraoperative optical coherence tomography in retinal surgery. Novel Diagn Methods Ophthalmol. 10.5772/intechopen.83511

